# Pharmacokinetics and Optimal Dosing of Levofloxacin in Children for Drug-Resistant Tuberculosis: An Individual Patient Data Meta-Analysis

**DOI:** 10.1093/cid/ciae024

**Published:** 2024-02-10

**Authors:** Yasmine N White, Belen P Solans, Paolo Denti, Louvina E van der Laan, H Simon Schaaf, Bryan Vonasek, Amyn A Malik, Heather R Draper, Hamidah Hussain, Anneke C Hesseling, Anthony J Garcia-Prats, Radojka M Savic

**Affiliations:** Department of Bioengineering and Therapeutics, Schools of Pharmacy and Medicine, University of California–San Francisco, San Francisco, California, USA; Department of Bioengineering and Therapeutics, Schools of Pharmacy and Medicine, University of California–San Francisco, San Francisco, California, USA; Center for Tuberculosis, University of California–San Francisco, San Francisco, California, USA; Division of Clinical Pharmacology, Department of Medicine, University of Cape Town, Cape Town, South Africa; Division of Clinical Pharmacology, Department of Medicine, University of Cape Town, Cape Town, South Africa; Desmond Tutu TB Centre, Department of Paediatrics and Child Health, Faculty of Medicine and Health Sciences, Stellenbosch University, Cape Town, South Africa; Desmond Tutu TB Centre, Department of Paediatrics and Child Health, Faculty of Medicine and Health Sciences, Stellenbosch University, Cape Town, South Africa; Department of Pediatrics, School of Medicine and Public Health, University of Wisconsin–Madison, Madison, Wisconsin, USA; TB Programs, Interactive Research Development (IRD) Global, Singapore, Singapore; Epidemiology department, Peter O'Donnell Jr. School of Public Health, UT Southwestern Medical Center, Dallas, Texas, USA; Desmond Tutu TB Centre, Department of Paediatrics and Child Health, Faculty of Medicine and Health Sciences, Stellenbosch University, Cape Town, South Africa; TB Programs, Interactive Research Development (IRD) Global, Singapore, Singapore; Desmond Tutu TB Centre, Department of Paediatrics and Child Health, Faculty of Medicine and Health Sciences, Stellenbosch University, Cape Town, South Africa; Desmond Tutu TB Centre, Department of Paediatrics and Child Health, Faculty of Medicine and Health Sciences, Stellenbosch University, Cape Town, South Africa; Department of Pediatrics, School of Medicine and Public Health, University of Wisconsin–Madison, Madison, Wisconsin, USA; Department of Bioengineering and Therapeutics, Schools of Pharmacy and Medicine, University of California–San Francisco, San Francisco, California, USA; Center for Tuberculosis, University of California–San Francisco, San Francisco, California, USA

**Keywords:** pediatrics, levofloxacin, pharmacokinetics, drug-resistant tuberculosis

## Abstract

**Background:**

Each year 25 000–32 000 children develop rifampicin- or multidrug-resistant tuberculosis (RR/MDR-TB), and many more require preventive treatment. Levofloxacin is a key component of RR/MDR-TB treatment and prevention, but the existing pharmacokinetic data in children have not yet been comprehensively summarized. We aimed to characterize levofloxacin pharmacokinetics through an individual patient data meta-analysis of available studies and to determine optimal dosing in children.

**Methods:**

Levofloxacin concentration and demographic data were pooled from 5 studies and analyzed using nonlinear mixed effects modeling. Simulations were performed using current World Health Organization (WHO)–recommended and model-informed optimized doses. Optimal levofloxacin doses were identified to target median adult area under the time-concentration curve (AUC)_24_ of 101 mg·h/L given current standard adult doses.

**Results:**

Data from 242 children (2.8 years [0.2–16.8] was used). Apparent clearance was 3.16 L/h for a 13-kg child. Age affected clearance, reaching 50% maturation at birth and 90% maturation at 8 months. Nondispersible tablets had 29% lower apparent oral bioavailability compared to dispersible tablets. Median exposures at current WHO-recommended doses were below the AUC target for children weighing <24 kg and under <10 years, resulting in approximately half of the exposure in adults. Model-informed doses of 16–33 mg/kg for dispersible tablets or 16–50 mg/kg for nondispersible tablets were required to meet the AUC target without significantly exceeding the median adult C_max_.

**Conclusions:**

Revised weight-band dosing guidelines with doses of >20 mg/kg are required to ensure adequate exposure. Further studies are needed to determine safety and tolerability of these higher doses.

## Teaser

Most children receiving levofloxacin for the treatment or prevention of rifampicin- and multidrug-resistant tuberculosis at currently recommended doses will not achieve adult-matched exposures. New guidelines with increased levofloxacin doses for children weighing <24 kg are proposed to ensure adequate exposure.

There are an estimated 1.2 million new cases of pediatric tuberculosis (TB) across the globe annually, with nearly half of these occurring in children aged <5 years [1–3]. Of these, approximately 3% are rifampin- or multidrug-resistant TB (RR/MDR-TB) [1]. Compared with adults, young children (aged <5 years) are at higher risk for TB disease progression and disseminated disease including TB meningitis [4]. For children with MDR-TB who are receiving appropriate treatment, mortality is 9%–12%, highlighting the importance of safe and effective therapies for children [5, 6].

The current World Health Organization (WHO) guidelines recommend the inclusion of a fluoroquinolone, either levofloxacin or moxifloxacin, in RR/MDR-TB treatment regimens for adults and children [2]. Fluoroquinolones have been used alone or in combination with other drugs for tuberculosis preventive treatment (TPT) in individuals exposed to RR/MDR-TB and are also conditionally recommended for this indication by WHO [7–9]. Preclinical and clinical studies suggest that levofloxacin and moxifloxacin are both effective in the treatment of TB, but levofloxacin has a more favorable cardiac and liver safety profile with extensive experience in young children, making it an attractive agent for use in combination with other QT-prolonging antimycobacterial or antiretroviral drugs [10–12]. Although preclinical studies in juvenile animals initially raised concern about musculoskeletal adverse effects of fluoroquinolones in children, extensive clinical studies of levofloxacin and other fluoroquinolones in children for a variety of indications have shown excellent safety in children with a wide range of infections, including long-term use in children with RR/MDR-TB [13–18]. Like moxifloxacin, levofloxacin is a concentration-dependent antibiotic with maximum concentration (C_max_) and area under the time-concentration curve (AUC) as primary determinants of safety and efficacy, respectively [19]. In contrast to moxifloxacin, levofloxacin is almost exclusively renally cleared, reducing the risk of hepatic drug–drug interactions [20]. For levofloxacin, mean C_max_ and AUC_24_ in healthy adults receiving the WHO-recommended dose of 750 mg daily are 11.9 mg/L and 101 mg·h/L, respectively [21].

Currently, WHO levofloxacin dosing recommendations consist of flat 15–20 mg/kg doses across all weight bands. This is inconsistent with the widely accepted nonlinear effects of allometry (body size) and maturation (age) on pharmacokinetics [22]. To achieve adequate levofloxacin exposures in children, one must suitably account for the rapid changes in organ maturity and body size. Lower fluoroquinolone concentrations may result in worse treatment outcomes with prolonged time to infection clearance and a higher rate of treatment failure [16, 23]. Several studies reporting the pharmacokinetics of levofloxacin in children have found suboptimal exposure in children compared with adult-matched exposure targets [24–29]. Studies of first-line treatments for TB, as well as moxifloxacin, have found that age, weight, and nutritional status can affect expected exposure in children [30–32]. Here, we perform an individual patient data meta-analysis (IPD-MA) of levofloxacin for the treatment and prevention of RR/MDR-TB in children using data from 4 previously published studies [26–29] and an additional study, MDR-PK2 for which linezolid and moxifloxacin, but not levofloxacin, data had been previously published, to identify covariates that affect exposure and derive more optimal dosing recommendations [30, 33].

## METHODS

### Clinical Studies and Data

We undertook a systematic review of studies reporting on levofloxacin pharmacokinetics in children and adolescents for any indication. Additional methods for identifying eligible studies are described in the Supplementary materials. Primary authors of eligible studies were asked to provide available patient-level information on demographics (age, sex, weight, height), clinical characteristics (human immunodeficiency virus [HIV] status, indication for anti-TB therapy), and levofloxacin administration details (drug dose, formulation, route of administration, dosing time, dosing interval, pharmacokinetic sampling time, plasma levofloxacin concentrations). Informed consent was obtained from all participants in the 5 studies for which deidentified data were used for the IPD-MA. This IPD-MA was approved by the Stellenbosch University Health Research Ethics Committee.

### Data Analyses

Levofloxacin concentration data were pooled and analyzed using nonlinear mixed-effects modeling with the software NONMEM and first-order conditional estimation with interaction for parameter estimation. Samples reported as below the limit of quantification were imputed at half the lower limit of quantification and included in the analysis. Additive, proportional, and combined error models were tested. Random effects at interindividual, interoccasion, and between-study variability were modeled exponentially. One- and 2-compartment models were evaluated with first-order absorption and linear and nonlinear elimination.

Model building was based on a likelihood ratio test, known as the objective function value (OFV), with a significance level of *P* < .05 and inspection of simulation-based diagnostics. Stepwise covariate modeling (*P* < .05 forward selection; *P* < .01 backward deletion) was performed to identify predictors of volume, clearance, bioavailability, and absorption, including weight, formulation, administration route (oral vs nasogastric tube), age, nutritional status (weight-for-age [WAZ], height-for-age [HAZ], weight-for-height [WHZ], and body mass index-for-age *z* scores [BMZ] based on WHO reference standards), HIV status, sex, and indication for treatment (TB disease or TPT). The final selection of the relevant covariates was guided by statistical significance and by clinical and physiological plausibility. Relative standard error (RSE) and 95% confidence interval for parameter estimates were determined using nonparametric bootstrapping with 500 samples.

### Exposure Evaluation

AUC_24,ss_ (24-hour AUC at steady state) was calculated as in Equation (1) (CL, clearance; F, apparent bioavailability) for all participants in the meta-analysis.


(1) $$\mathrm{AU}C_{24,\mathrm{ss}}=\frac{\mathrm{Dose}\cdot F}{\mathrm{CL}}$$

Steady-state pharmacokinetics were simulated 500 times using the aggregated pediatric TB population from the included studies using current WHO weight band dosing and compared with the median AUC_24,ss_ and C_max_ in adults following a standard 750-mg dose once daily using 250-mg nondispersible levofloxacin tablets (AUC_24,ss_ of 101 mg × h/L and C_max_ 11.9 mg/L [21]).

### Dose Optimization

Optimal dosing of levofloxacin using either commercially available 100-mg dispersible tablets or 250-mg nondispersible tablets was calculated targeting AUC_24,ss_ of 101 mg × h/L using the final model developed above. Optimal doses were rounded to half-tablet increments, when possible, for both formulations, and the current WHO dosing weight bands were retained. Steady-state pharmacokinetics were again simulated 500 times as described above, and exposure parameters were compared to adult standards.

### Statistics and Software

NONMEM 7.5.1 and Perl-speaks NONMEM 4.8.1 were used for modeling and simulation. Selection between models was based on the minimum value of the objective function provided by NONMEM, which is equal to −2 × log likelihood (−2LL). For nested models that differed in 1 parameter, −2LL differences of 3.84 (*P* = .05) were considered significant. Visual diagnostics were performed with “Xpose4” (4.7.2) and “vpc” (1.2.1) R packages in R 4.2.0. Models were diagnosed using visual exploration of the goodness-of-fit plots and visual predictive checks. Parameter precision was further evaluated through 500 nonparametric bootstraps.

## RESULTS

### Patients and Sampling

Five studies were identified for inclusion in this analysis (see Supplementary materials). Details are shown in Table 1. In all studies, pharmacokinetic sampling was performed after at least 7 days of levofloxacin dosing. Patient characteristics for the 242 children with available pharmacokinetic data are summarized in Table 2. Thirty-one children contributed pharmacokinetic samples on more than 1 occasion. Seven samples were excluded from the meta-analysis due to levels and pharmacokinetic profile inconsistent with dosing time. Median WAZ was below zero for all studies, and 28% of children had at least 1 nutrition parameter with a *z* score < −2 (WAZ, HAZ, WHZ, and BMZ). Levofloxacin concentrations were below the lower limit of quantification in 66 of the 1475 samples tested across all studies (4.5%), mostly in predose samples.

**Table 1.** Studies Included in Individual Patient Data Meta-Analysis and Methodology of Included Studies

	Author and Year				
	Malik et al, 2019 [26]	Denti et al, 2018 [27]	Garcia-Prats et al, 2019 [28]	Van der Laan et al, 2023 [29]	MDR-PK2
Brief study description	PK of nondispersible levofloxacin tablets	PK and safety of levofloxacin and other second-line anti-TB drugs at routine doses	PK of levofloxacin dispersible tablets	Comparison of PK of dispersible and nondispersible tablets using a crossover design	PK and safety of moxifloxacin, levofloxacin, and linezolid
Country and sample size	Pakistan n = 24	South Africa n = 109	South Africa n = 24	South Africa n = 25	South Africa n = 60
Dosing regimen	≤5 y, 15–20 mg/kg; >5 y, 7.5–10 mg/kg	15–20 mg/kg	15–20 mg/kg	15–20 mg/kg	15–25 mg/kg
Formulation	250- or 500-mg nondispersible tablets whole or crushed	250-mg nondispersible tablet whole or crushed	100-mg dispersible tablets	100-mg dispersible tablets and crushed 250-mg nondispersible tablets	250-mg nondispersible tablet whole or crushed
Lower limit of quantification^a^	0.20 mg/L	0.0781 mg/L	0.0781 mg/L	0.0781 mg/L	0.0781 mg/L
Indication for treatment	TPT	MDR-TB treatment, TPT	TPT	TPT	MDR-TB treatment
Timing of sampling	>1 m on therapy; 0, 1, 2, and 6 h post dose	Between 2 and 16 wk on therapy; 0, 1, 2, 4, 6, and 8 h post dose	7–14 d on therapy; 0, 1, 2, 4, 6, and 8 h post dose	>2 wk on therapy; 0, 1, 2, 4, 6, and 8 h post dose	>2 wk on therapy; 0, 1, 4, and 10 h post dose

Abbreviations: MDR-TB, multidrug-resistant tuberculosis; PK, pharmacokinetics; TB, tuberculosis; TPT, tuberculosis preventive therapy.

^a^Method of quantification: liquid chromatography-tandem mass spectrometry for all studies.

**Table 2.** Demographic and Clinical Characteristics of Children Treated With Levofloxacin

	Author and Year, Sample Size					
	Malik et al 2019, N = 24 [26]	Denti et al 2018, N = 109 [27]	Garcia-Prats et al 2019, N = 24 [28]	Van der Laan et al 2023 N = 25 [29]	MDR-PK2, N = 60	Total n = 242
Living with human immunodeficiency virus, n (%)	Not available	16 (15)	0 (0)	0 (0)	4 (7)	20 (8)
Male sex, n (%)	17 (71)	56 (51)	15 (63)	15 (60)	28 (47)	131 (54)
Age, median (range), y	5.0 (2.0, 10.0)	2.1 (0.3, 8.7)	2.1 (0.5, 4.6)	2.6 (0.2, 6.0)	4.1 (0.8, 16.3)	2.8 (0.2, 16.3)
Weight, median (range), kg	16.5 (7.0, 27.0)	12.4 (5.9, 21.8)	12.3 (6.4, 14.9)	12.2 (4.0, 20.3)	13.8 (7.6, 48.5)	12.9 (4.0–48.5)
WAZ,^a^ median (range)	−1.1 (−4.6, 0.8)	−0.4 (−4.0, 3.4)	−0.7 (−4.0, 1.4)	−0.6 (−2.5, 2.0)	−0.8 (−4.0, 1.4)	−0.6 (−4.6, 3.4)
HAZ,^a^ median (range)	−0.9 (−3.6, 1.1)	−1.3 (−4.7, 1.5)	−0.7 (−3.9, 1.1)	−1.2 (−3.6, 1.0)	−1.1 (−4.0, 2.2)	−1.1 (−4.7, 2.2)
WHZ,^a^ median (range)	−1.5 (−4.2, 0.3)	0.5 (−4.6, 4.5)	0.0 (−3.3, 2.0)	0.2 (−1.8, 2.2)	−0.1 (−2.2, 1.8)	0 (−4.6, 4.5)
BMZ, median (range)	−1.1 (−3.8, 0.5)	0.7 (−4.7, 4.4)	−0.2 (−3.0, 2.2)	0 (−1.6, 2.1)	−0.1 (−3.2, 2.0)	0.2 (−4.7, 4.4)
Malnourished,^b^ n (%)	9 (38)	35 (32)	5 (21)	6 (24)	13 (22)	68 (28)

Abbreviations: BMZ, body mass index-for-age *z* score; HAZ, height-for-age *z* score; WAZ, weight-for-age *z* score; WHZ, weight-for-height *z* score.

^a^For those aged <5 years.

^b^Defined as WAZ, HAZ, WHZ, or BMZ < −2.

### Population Pharmacokinetics

**Figure 1.**
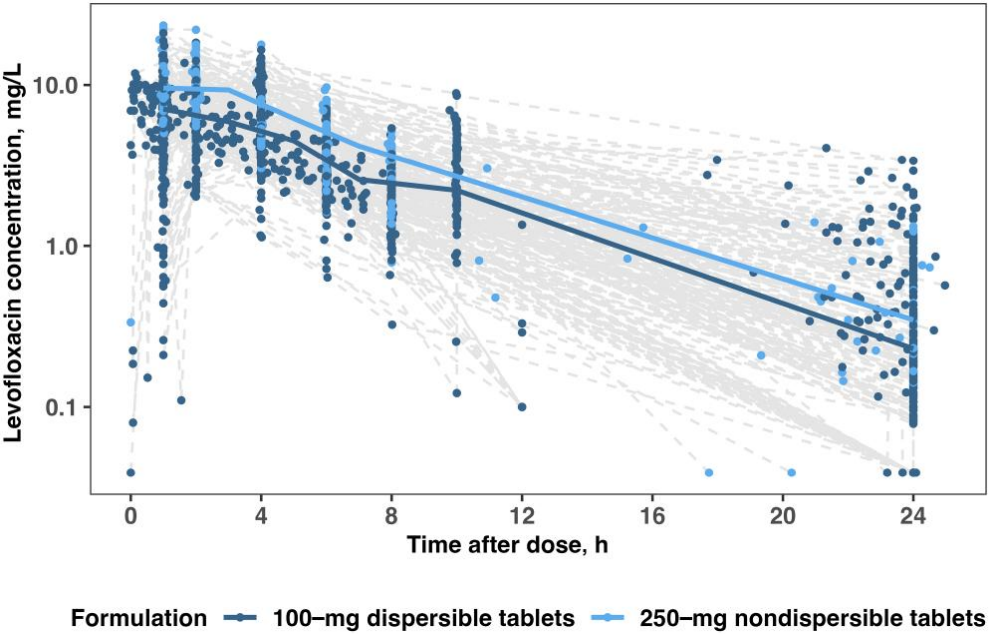
Levofloxacin pharmacokinetic profiles in children included in individual patient data meta-analysis stratified by formulation. Dashed lines connect individual observed concentrations.

Levofloxacin pharmacokinetic profiles were notable for slightly higher plasma concentrations in children who received dispersible tablets but were otherwise similar between studies (Figure 1, Supplementary Figure 1). The population pharmacokinetics were best described with 1-compartment distribution with first-order absorption with a lag time and linear elimination. Although addition of a second distribution compartment improved the OFV, parameter estimates were imprecise. The model diagnostics did not show significant improvement; therefore, a 1-compartment model was chosen for the final model structure.

Allometric scaling of volume of distribution and clearance by body weight improved the model fit. Apparent clearance or bioavailability did not differ significantly by nutritional status (WAZ, HAZ, WHZ, and BMZ as continuous covariates or the presence of any *z* score <−2 as a categorical covariate) or indication for treatment (preventive treatment or disease). Children with HIV did not have significantly different clearance compared with children without HIV. Relative bioavailability of nondispersible 250-mg tablets was 29% lower than for dispersible 100-mg tablets. Absorption was not significantly affected by administration with nasogastric tube, although these data were missing for 10% of participants.

The inclusion of maturation of CL with age improved the model fit, and the value of 8.94 for post-menstrual age at which 50% maturation is reached (PMA_50_) was consistent with previously reported values for renally cleared therapeutics [34].

**Figure 2.**
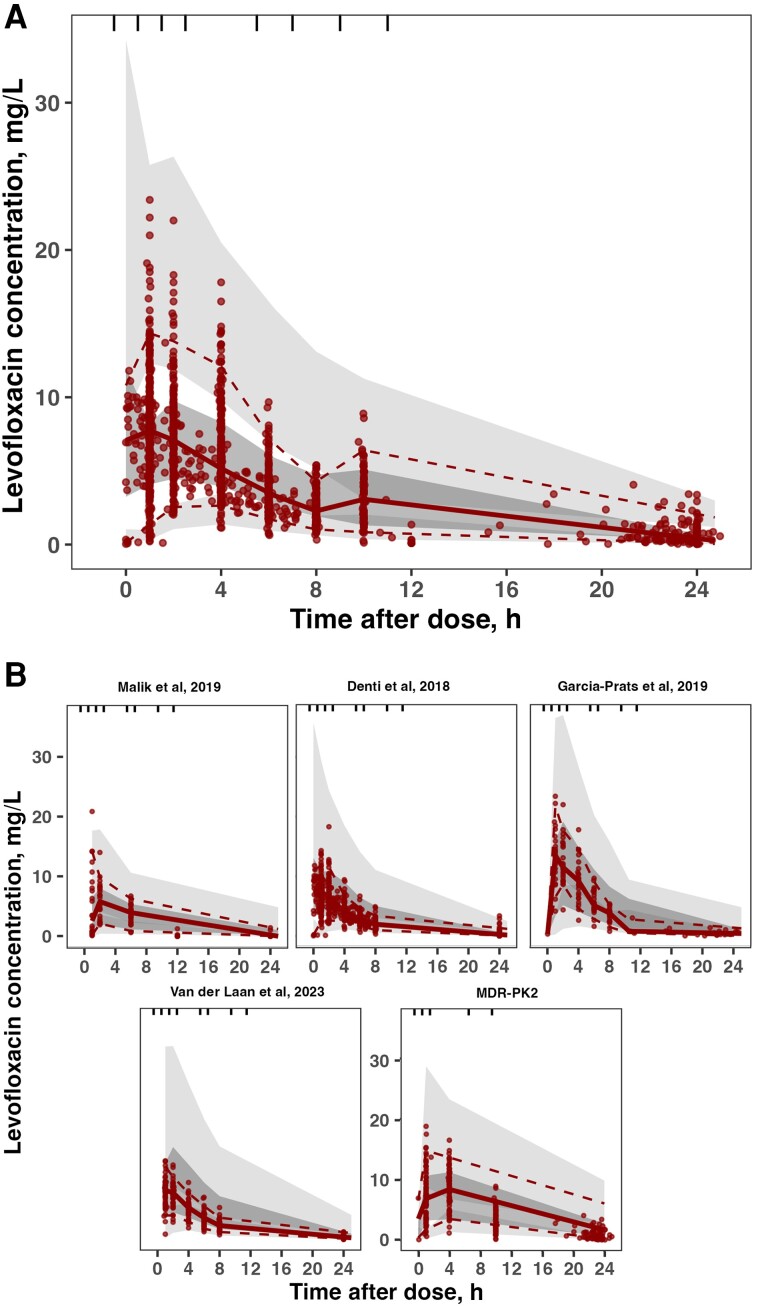
Visual predictive check of the final pharmacokinetic model for the individual patient data meta-analysis population (*A*) and stratified by study (*B*). Dots represent observed data. Lines correspond to the 5th (dashed), 50th (solid), and 95th (dashed) percentiles of observed data. Shaded areas are the model-predicted 95% confidence intervals for the 5th, 50th, and 95th percentiles obtained from the 500 simulated datasets. Abbreviation: MDR-PK2, multidrug-resistant.

**Figure 3.**
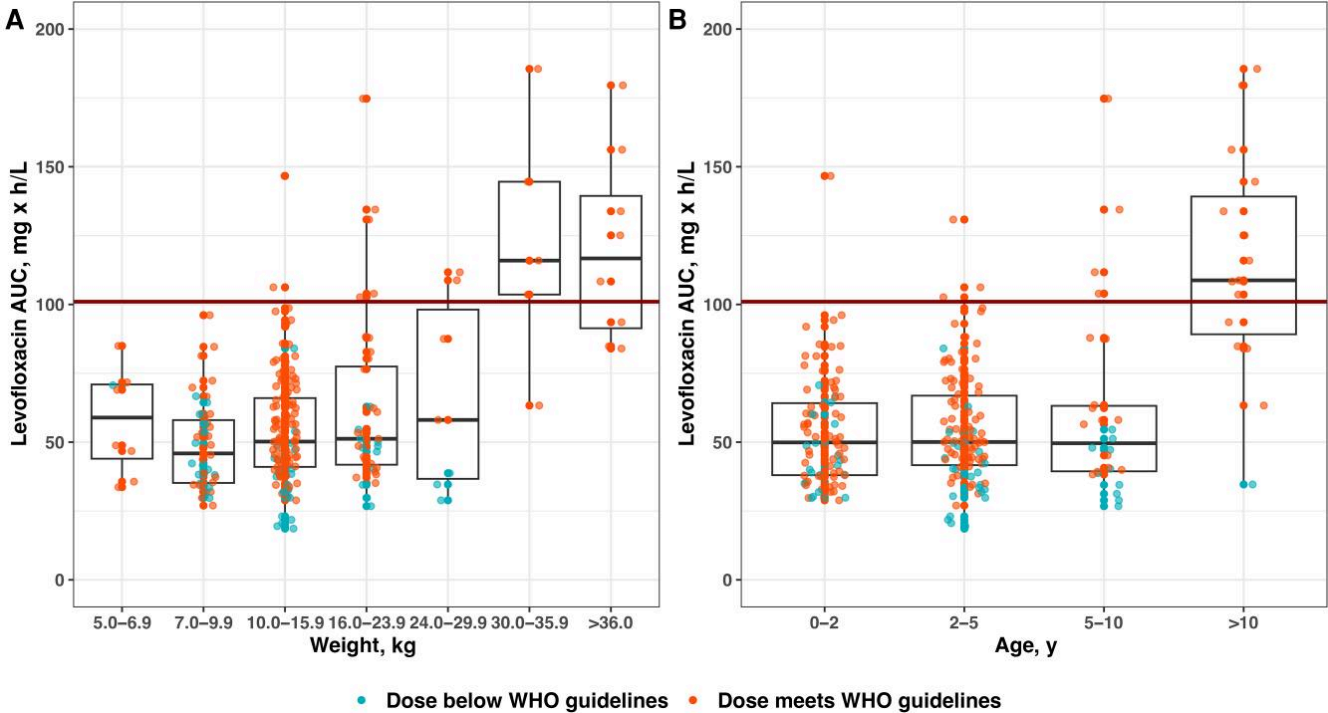
Observed levofloxacin AUC_24_ at steady state for all patients stratified by weight (*A*) and stratified by age (*B*). Abbreviation: AUC, area under the time-concentration curve; WHO, World Health Organization.

Table 3 shows the final pharmacokinetic parameter estimates. The pharmacokinetic model predicted the observed data well (Figure 2, Supplementary Figure 1). The median AUC_24_ (2.5%, 97.5%) of all included individual dosing occasions was 51.2 mg × h/L (26.9 mg × h/L, 136 mg × h/L) and was lowest in children who weighed ≤24 kg or were aged ≤10 years (Figure 3).

**Table 3.** Population Pharmacokinetic Parameter Estimates in Children Receiving Levofloxacin for Rifampicin- or Multidrug-Resistant Tuberculosis Preventive Therapy or Treatment

Parameter	Population Estimate (Relative Standard Error %)	Bootstrap 95% Confidence Interval
CL (L/h)^a,b^	3.16 (4)	3.02–3.42
V (L)^c^	17.0 (4)	15.5–18.7
K_a_ (1/h)	2.78 (9)	2.36–3.51
ALAG (h)	0.55 (7)	.44–.60
F dispersible tablet	1.0 (fixed, reference)	…
Covariate effects	…	…
Nondispersible tablet formulation on F (%)	−28.7 (12)	−37.8–−21.1
Maturation function for CL—PMA_50_ (mo)^b^	8.94 (8)	7.94–11.1
Maturation function for CL—Hill coefficient (.)^b^	3.46 (18)	2.88–5.01
Interindividual variability (%)	…	…
CL	19.89 (12)	17.6–22.7
Interoccasion variability (%)	…	…
K_a_	161 (13)	131–178
F	32.9 (19)	27.1–38.6
ALAG	33.7 (31)	29.7–43.3
Study-level random effects (%)	…	…
CL	13.3 (14)	10.5–15.0
F	30.4 (14)	22.8–33.1
Random effects	…	…
Additive error (mg/L)	0.19 (7)	.17–.22
Proportional error (%)	14.2 (8)	11.4–16.3

Abbreviations: ALAG, latency time; CL, clearance; F, bioavailability; K_a_, absorption rate constant; PMA_50_, post-menstrual age at which 50% maturation is reached; TM_50_, maturation half time; V, volume of distribution.

^a^Allometrically scaled to median weight of population [13 kg] with exponent of 0.75 for CL and 1 for V.

^b^ $\mathrm{CL}=\theta_{\;pop}\times {\left(\frac{WT}{13}\right)}^{0.75}\;\times \;\frac{PMA^{Hill}}{TM_{50}^{Hill}+PMA^{Hill}}$.

^c^ $V=\theta_{\;pop}\times \left(\frac{WT}{13}\right)$

### Optimal Dosing Simulations

**Figure 4.**
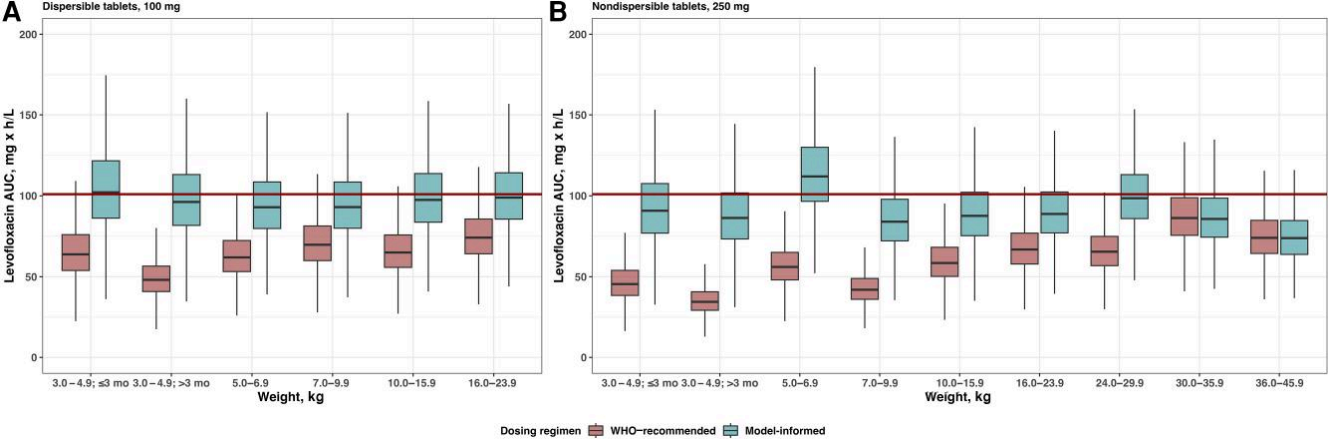
Simulated levofloxacin AUC_24_ at steady state according to current WHO recommendations and with model-informed optimized doses. AUC_24, ss_ for dosing of 100-mg dispersible tablets (*A*) or 250-mg nondispersible tablets (*B*). Data are based on 500 simulations. Abbreviations: AUC, area under the time-concentration curve; WHO, World Health Organization.

Model-informed optimized and practically implementable doses (rounded to the nearest half-tablet increment) were 16–50 mg/kg of the 250-mg nondispersible levofloxacin tablets for children who weighed 3–46 kg and 16–33 mg/kg of the 100-mg dispersible levofloxacin tablets for children who weighed 3–24 kg, with lighter patients requiring the highest milligram per kilogram dose. Model-informed dosing resulted in higher predicted exposures approaching the target AUC_24_ of 101 mg·h/L based on typical adult exposure with predicted C_max_, similar to that observed in adults who received a standard 750-mg dose of nondispersible levofloxacin (Figure 4, Supplementary Figure 2). Model-informed dosing was up to double the current WHO-recommended dosing with the largest increases in the lightest weight bands (Table 4).

**Table 4.** Currently Recommended and Optimized Pediatric Weight-Banded Dosing for Levofloxacin

	Current World Health Organization Dosing^a^				Model-Informed Optimized Dosing			
	100-mg Dispersible Tablet		250-mg Tablet		100-mg Dispersible Tablet		250-mg Tablet^b^	
Weight Band	Tablets	Dose, mg	Tablets	Dose, mg	Tablets	Dose, mg	Tablets	Dose, mg
3.0–4.9 kg, <3 mo	0.5 (5 mL)	50	0.2 (2 mL)	50	0.8 (8 mL)^b^	80^b^	0.4 (4 mL)^c^	100^c^
3.0–4.9 kg, >3 mo	0.5 (5 mL)	50	0.2 (2 mL)	50	1	100	0.5 (5 mL)^c^	125^c^
5.0–6.9 kg	1	100	0.5	125	1.5	150	1	250
7.0–9.9 kg	1.5	150	0.5	125	2	200	1	250
10.0–15.9 kg	2	200	1	250	3	300	1.5	375
16.0–23.9 kg	3	300	1.5	375	4	400	2	500
24.0–29.9 kg	…	…	2	500	…	…	3	750
30.0–35.9 kg	…	…	3	750	…	…	3	750
36.0–45.9 kg	…	…	3	750	…	…	3	750

^a^Based on World Health Organization 2022 guidelines [2].

^b^Dosing with 100-mg dispersible tablets is preferred for children who weigh <24 kg.

^c^For infants who weigh 3–5 kg and are aged <3 months receiving dispersible tablets and all infants who weigh 3–5 kg receiving nondispersible tablets, the indicated volume of an extemporaneous suspension made by crushing and dissolving 1 tablet in 10 mL of water is recommended.

## DISCUSSION

This study provides an IPD-MA of the pharmacokinetics of levofloxacin in a large number of well-characterized children who received treatment for RR/MDR-TB exposure or disease. Levofloxacin exposures in children who received the current WHO-recommended doses were lower than the target exposure extrapolated from adults who received a 750-mg daily dose across all studies, especially for younger and smaller children, placing them at higher risk for unsuccessful treatment. Dispersible levofloxacin tablets had higher bioavailability compared with nondispersible tablets and also allowed for more precise dosing. However, the proposed model-optimized doses, which would achieve adequate exposure in all children and align with current WHO weight bands, can be practically implemented with both available oral formulations.

Our study showed that levofloxacin exposure in children aged <10 years or who weighed <24 kg was substantially lower than the target exposure derived from adults who received a standard daily dose of 750 mg. Failure to meet the adult levofloxacin exposure median places children at higher risk for unfavorable treatment outcomes. Our model structure did not significantly differ from previously published models of levofloxacin pharmacokinetics in children, and we consistently report that exposure in children is below the target extrapolated from adults and similar model-informed dosing recommendations [26–29]. It is also important to highlight that even though there were 20 children (8%) with HIV, an effect of the disease or of concomitant administration of antiretrovirals could not be identified in the model. Similarly, even though there is substantial representation of malnourished children, an effect of malnutrition (in addition to the effects of weight and age already included in the model) could not be identified.

The predicted exposure across all weight bands was improved using model-informed dosing of 16–33 mg/kg of dispersible tablet formulations so that projected median exposure would be approximately 101 mg × h/L. This represents an increase of up to 50% in doses compared with the current WHO dosing recommendations of dispersible tablets and should therefore be implemented with caution. Adequate exposure can also be achieved using nondispersible tablets, although projected median exposure in the lowest weight bands (3–4.9 kg, 5.0–6.9 kg) would be well above or below the target if doses were rounded to the nearest half tablet, increasing the risk of poor outcomes in this younger age group. Similarly, although the rounded model-derived dose of nondispersible tablets results in projected exposure that is slightly below the target for 7–9.9 kg, 10–15.9 kg, and 16–23.9 kg weight bands, increasing the dose for each weight band by 0.5 tablets resulted in projected exposure and C_max_ well above the adult median, a recommendation that is not yet supported by adequate safety data. Given the difficulty of achieving target exposures in the 3–4.9 kg and 5.0–6.9 kg weight bands with 250-mg nondispersible tablets, use of the child-friendly 100-mg dispersible tablet formulation in this weight band is strongly preferred when available. If 100-mg dispersible tablets are not available, we recommend dissolving a single 250-mg nondispersible levofloxacin tablet in a small volume of water, for example, 10 mL, and to allow for administration of more precise doses (Table 4). This method is not preferred due to its complexity and potential for medication administration errors. Similarly, in children who weigh <24 kg, the use of the child-friendly 100-mg dispersible tablet formulation would be preferred since the exposures would be more optimal. The safety of implementing treatment regimens that use higher doses of levofloxacin is supported by the model-predicted C_max_, which aligns with the median C_max_ in adults following standard 750-mg daily dosing and is below the C_max_ in adults following a higher 1000-mg dose [21]. Additionally, in a small study in Thailand in which 5 children with TB were treated with doses above the current WHO recommendation (20–30 mg/kg; median, 25 mg/kg), no QT prolongation or other grade 3 or 4 adverse events were observed [35]. However, larger prospective studies are needed to definitively assess the safety of the higher proposed doses of levofloxacin in children, both for prevention and treatment.

Additionally, the implications of the results of this IPD-MA for levofloxacin dosing for children receiving RR/MDR-TB TPT will need to be carefully considered in light of results from trials of levofloxacin efficacy and safety for this indication. The TB-CHAMP (ISRCTN92634082) phase 3 trial tested 15- to 20-mg/kg doses for RR/MDR-TB TPT in children, using more conservative doses in well children than what we are suggesting for TB treatment. Results from this trial have shown that it was effective in preventing TB disease and was very safe and well tolerated, suggesting that lower doses are effective for preventive therapy [36, 37]. Additional results from V-QUIN (ACTRN1261600021542) for adults and adolescents also support the use of levofloxacin in an effective and safe manner for TB prevention [38, 39].

A limitation of our study is the relatively small number of children at the low and high end of the weight and age ranges. Ten infants aged <6 months and 1 who weighed <5 kg were included, which, although modest in number, is still large compared with participant data available from individual studies and for other second-line TB drugs in children. Because of this, model-predicted exposures and dosing recommendations for infants and children who weigh <5 kg should be implemented with caution until additional studies in this population can be completed. However, given the need for guidance for the clinician tasked with treating these children, and with consideration of spectrum and severity of TB disease observed, we provide the best estimates with the currently available data. Similarly, only 13 children who weighed >30 kg were included, limiting the ability of the study to delineate at what point bioavailability and other parameters normalize to adult values. Although 242 children were included in this meta-analysis, the number of children with HIV (n = 20) and the number of malnourished children were relatively small (n = 68 with at least 1 *z* score <−2), limiting the power to detect differences in exposure based on these characteristics, which have been associated with increased clearance in other pediatric studies [27, 30]. Additionally, although 5 distinct studies were included in this analysis, they were all conducted in either South Africa or Pakistan, which may limit generalizability. However, a previous study of children in the Federated States of Micronesia and the Republic of the Marshall Islands also predicted an AUC_24_ below our target of 101 mg·h/L with doses up to 20 mg/kg [25]. A final limitation is the lack of patient-level pharmacodynamic data that included TB treatment outcomes. Pediatric-specific pharmacodynamic studies are not available to directly support an exposure target in children. In this setting, European and American regulatory bodies recommend extrapolation of adult exposure targets to children on the assumption that exposure–response relationships should be consistent across age groups for the same disease, as we have done here [40, 41].

In conclusion, we believe that our individual patient data meta-analysis supports the revision of current pediatric levofloxacin dosing guidelines for MDR-TB treatment and prevention to ensure adequate exposure in all children using existing formulations and without compromising patient safety.

## Supplementary Data

Supplementary materials are available at *Clinical Infectious Diseases* online. Consisting of data provided by the authors to benefit the reader, the posted materials are not copyedited and are the sole responsibility of the authors, so questions or comments should be addressed to the corresponding author.

## Supplementary Material

ciae024_Supplementary_Data

## Notes

***Author Contributions.*** Y. N. W., B. P. S., and R. M. S. contributed to the analysis plan. H. S. S., L. V. D. L., B. V., A. A. M., H. R. D., H. H., A. C. H., A. J. G. P., and P. D. contributed to preparation of individual study data for analysis. Y. N. W. and B. P. S. performed the modeling and simulation and prepared the manuscript with input and support from A. J. G. P., P. D. and R. M. S. All authors reviewed and approved the final manuscript prior to submission.

***Acknowledgments.*** The authors thank all the children, families, caregivers, and healthcare workers who participated in the studies included in this meta-analysis.

***Financial support*.** This work was supported by Unitaid funding and support through the BENEFIT Kids project awarded to Stellenbosch University. Y. N. W. was supported by National Institutes of Health grant T32GM007546.
