# Supplementary material for: Pharmacokinetics and Optimal Dosing of Levofloxacin in Children for Drug-Resistant Tuberculosis: An Individual Patient Data Meta-Analysis
Source: Clin Infect Dis. 2024 Feb 10;78(3):756–64. doi: 10.1093/cid/ciae024 (PMC10954342; doi:10.1093/cid/ciae024)
Supplement: ciae024_Supplementary_Data [file ciae024_supplementary_data.zip › SupplementaryMaterial.docx]

**Supplementary Material**

**Identification and selection of eligible studies**

This systematic review and individual participant data meta-analysis of the pharmacokinetics of second-line antituberculosis drugs in children is registered with PROSPERO, with the protocol available at: <https://www.crd.york.ac.uk/PROSPEROFILES/187260_PROTOCOL_20200520.pdf>

Briefly, we conducted a comprehensive search for all relevant evidence, regardless of language or publication status in the following databases: PubMed, Scopus, and CENTRAL databases through 30 April 2020. Search terms included both exploded medical subject headings (MeSH) and free-text terms (see protocol for detailed search strategy). Colleagues of the authors and known experts in the field were also asked to provide any eligible unpublished data.

Two authors independently screened titles and abstracts recovered from the database search. Full-text articles were sought for records considered potentially eligible by both authors. Both authors then independently reviewed the full-text articles for eligibility. For any disagreement between the two authors about the eligibility of a full-text article, a third author resolved the disagreement. If missing information in the full-text article resulted in unclear eligibility, the authors of the primary article were contacted for clarification.

A study cohort was eligible for inclusion if it included individuals <20 years old at the time of treatment with levofloxacin for any indication and reported on levofloxacin pharmacokinetics. Eligibility criteria were applied at the individual level, allowing for the potential inclusion of studies that also included individuals 20 years and older. Eligible individual patient data was required to include drug concentrations with time relative to dosing, use of a validated assay for drug concentration quantification with reported lower limit of quantification, levofloxacin treatment history (duration, route, dose, frequency) prior to sampling for drug concentrations, and patient age and weight. Data from unpublished studies were included if data collection was approved by the ethics board of the originating institution and appropriate permissions were in place to share the data. There were no restrictions on study design or dates of data collection or publication.

For eligible studies identified, authors were contacted to request individual patient data. With written agreement from the original authors (or the principal investigator for studies of unpublished data), de-identified data were obtained for meta-analysis.

**Results of search and selection of included studies**

The systematic review identified 8 potentially eligible studies. The original authors of 4 published studies and 1 unpublished study were contacted, agreed to share individual patient data, and the studies were included in the meta-analysis as described in the main manuscript. Three other studies were identified but the original authors did not respond to requests to share data (1, 2) or were unable to share the individual data (3). This included studies of levofloxacin pharmacokinetics from one study in 25 children treated for MDR-TB (1), one study in 50 children in the Federated States of Micronesia and The Republic of the Marshall Islands receiving levofloxacin preventive therapy for MDR-TB (3), and one study of single doses of levofloxacin in 85 children in the United States with pneumonia (2).

**References**

1. Hemanth Kumar AK, Kumar A, Kannan T, Bhatia R, Agarwal D, Kumar S, Dayal R, Singh SP, Ramachandran G. 2018. Pharmacokinetics of Second-Line Antituberculosis Drugs in Children with Multidrug-Resistant Tuberculosis in India. Antimicrob Agents Chemother 62.

2. Chien S, Wells TG, Blumer JL, Kearns GL, Bradley JS, Bocchini JA, Jr., Natarajan J, Maldonado S, Noel GJ. 2005. Levofloxacin pharmacokinetics in children. J Clin Pharmacol 45:153-60.

3. Mase SR, Jereb JA, Gonzalez D, Martin F, Daley CL, Fred D, Loeffler AM, Menon LR, Bamrah Morris S, Brostrom R, Chorba T, Peloquin CA. 2016. Pharmacokinetics and Dosing of Levofloxacin in Children Treated for Active or Latent Multidrug-resistant Tuberculosis, Federated States of Micronesia and Republic of the Marshall Islands. Pediatr Infect Dis J 35:414-21.

**
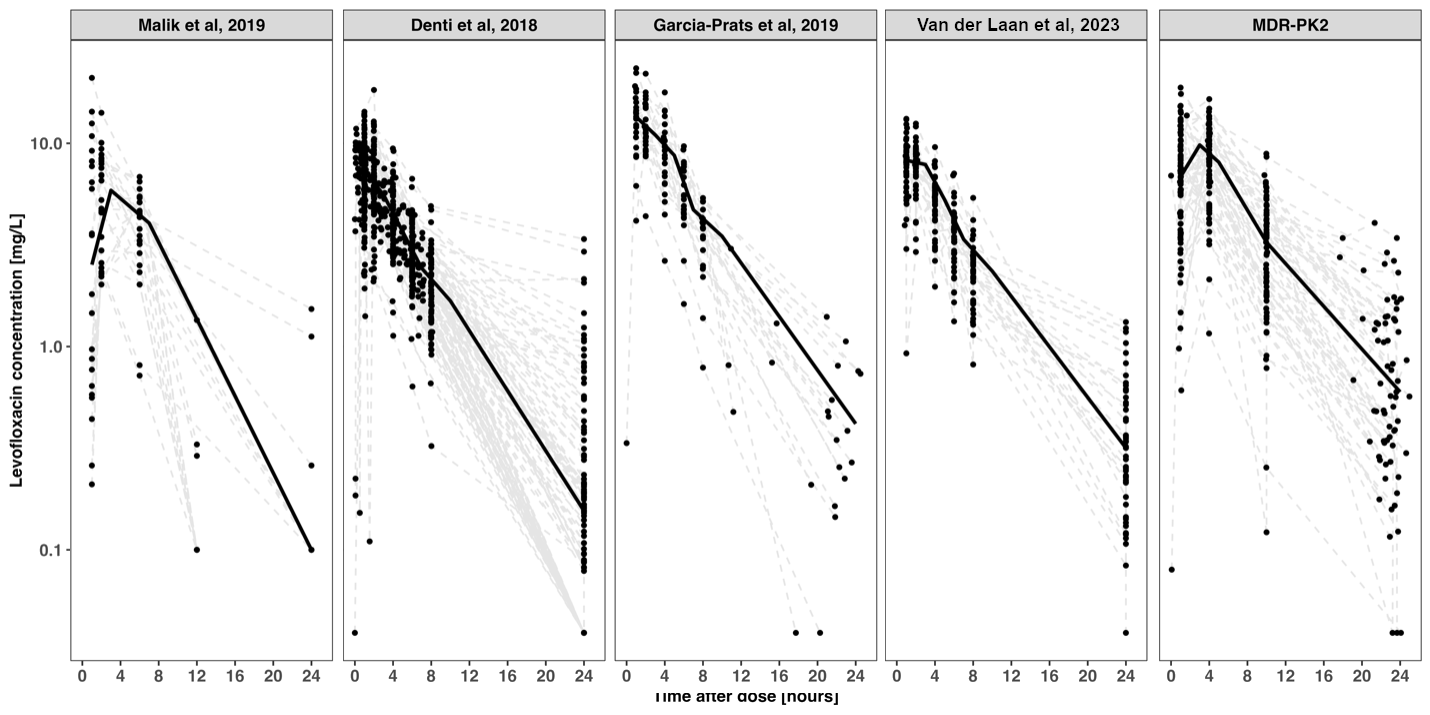
**

**Supplemental Figure 1.** Levofloxacin pharmacokinetic profiles in children stratified by study. Gray lines connect individual observed concentrations.

**
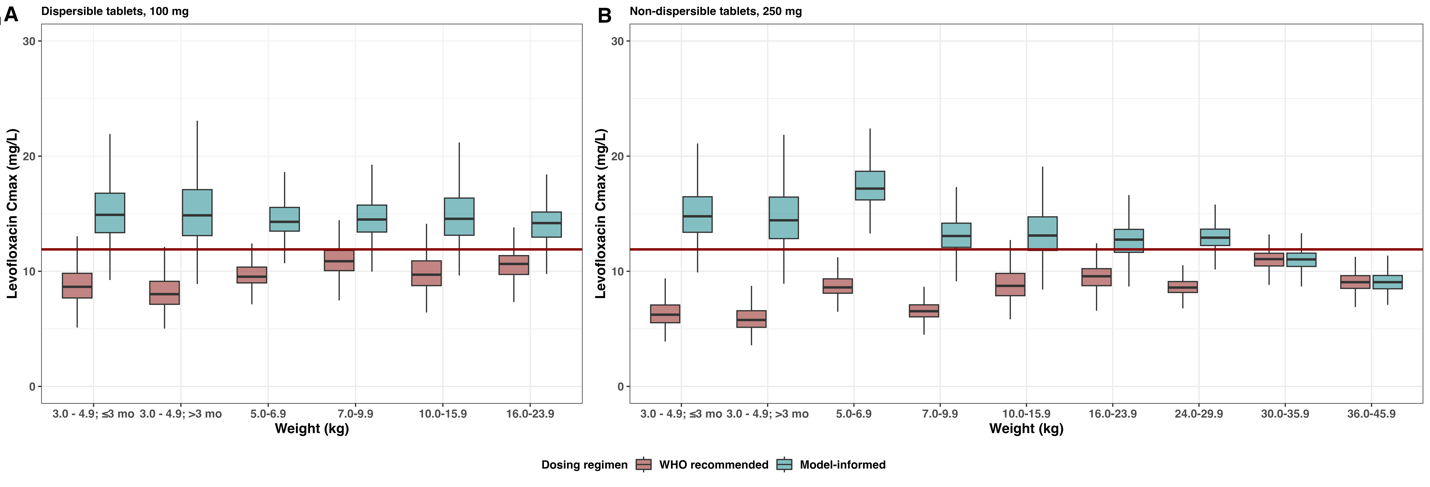
Supplemental Figure 2. Simulated levofloxacin C_max_ at steady state according to current WHO recommendations and with model-informed optimized doses.** Cmax for dosing of 100 mg dispersible tablets (A) or 250 mg non-dispersible tablets (B). Data are based on 500 simulations. Solid line represents target C_max_. Model optimized doses are as listed in Table 4.
