# Supplementary figures and images for: Pharmacokinetics and Optimal Dosing of Levofloxacin in Children for Drug-Resistant Tuberculosis: An Individual Patient Data Meta-Analysis
Source: Clin Infect Dis. 2024 Feb 10;78(3):756–64. doi: 10.1093/cid/ciae024 (PMC10954342; doi:10.1093/cid/ciae024)

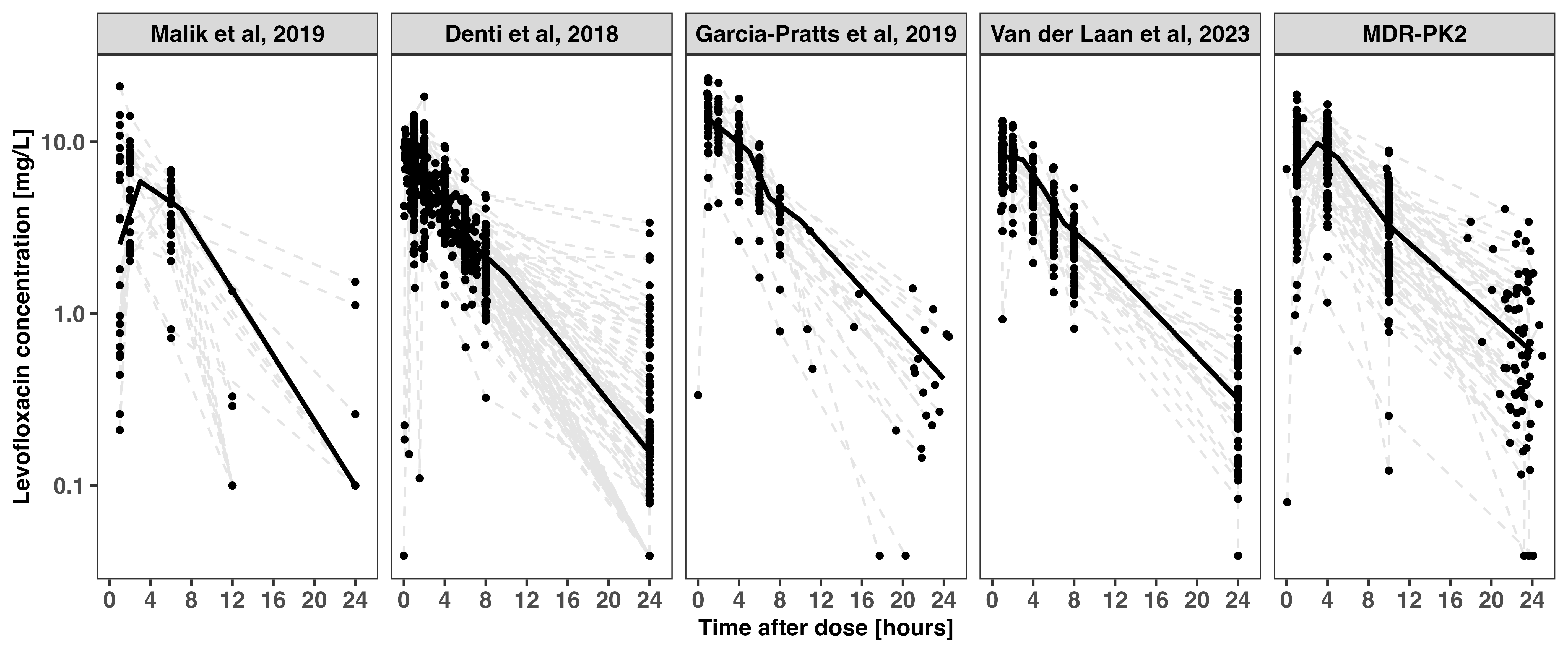

Supplement: ciae024_Supplementary_Data [file ciae024_supplementary_data.zip › SupplFigure1.tiff]
